# Modeling of Nanoparticular Magnetoresistive Systems and the Impact on Molecular Recognition

**DOI:** 10.3390/s150409251

**Published:** 2015-04-20

**Authors:** Lisa Teich, Daniel Kappe, Thomas Rempel, Judith Meyer, Christian Schröder, Andreas Hütten

**Affiliations:** 1 Bielefeld Institute for Applied Materials Research, Computational Materials Science and Engineering, University of Applied Sciences Bielefeld, Wilhelm-Bertelsmann-Str. 10, Bielefeld 33602, Germany; E-Mails: lisa.teich@fh-bielefeld.de (L.T.); dkappe@physik.uni-bielefeld.de (D.K.); christian.schroeder@fh-bielefeld.de (C.S.); 2 Center for Spinelectronic Materials and Devices, Department of Physics, Bielefeld University, P.O. 100131, Bielefeld 33501, Germany; E-Mails: trempel@physik.uni-bielefeld.de (T.R.); judith.mey@gmx.de (J.M.)

**Keywords:** hybrid combination of classical spin dynamics and molecular dynamics simulations, nanoparticular GMR-effect, sensor based determination of association and dissociation constants in molecular recognition

## Abstract

The formation of magnetic bead or nanoparticle superstructures due to magnetic dipole dipole interactions can be used as configurable matter in order to realize low-cost magnetoresistive sensors with very high GMR-effect amplitudes. Experimentally, this can be realized by immersing magnetic beads or nanoparticles in conductive liquid gels and rearranging them by applying suitable external magnetic fields. After gelatinization of the gel matrix the bead or nanoparticle positions are fixed and the resulting system can be used as a magnetoresistive sensor. In order to optimize such sensor structures we have developed a simulation tool chain that allows us not only to study the structuring process in the liquid state but also to rigorously calculate the magnetoresistive characteristic curves for arbitrary nanoparticle arrangements. As an application, we discuss the role of magnetoresistive sensors in finding answers to molecular recognition.

## 1. Introduction

The giant magnetoresistance (GMR) effect was originally discovered in magnetic multilayer systems in 1988/89 [1,2] but it was soon extended to granular systems [3,4], *i.e.*, samples based on magnetic nanoparticles in metallic matrices. By dispersing cobalt nanoparticles in conductive gel-like matrices, it was shown [5] that very high GMR effect amplitudes of up to 260% can be realized which makes it an interesting candidate for printable magnetoresistive sensor devices. In order to obtain high GMR effect amplitudes the gel matrix needs to have a high viscosity and a high electrical conductivity. This in combination leads to an increased particle density along the current path by the formation of particle chains. Furthermore, magnetoresistive measurements show that ionic mobility is crucial for the effect amplitude as well. Moreover, 3D reconstructions of samples have shown that the close proximity of particles and clusters plays an important role. Tuning the magnetoresistive properties is very important to achieve a sensor device with optimal sensitivity. In this article we present a tool chain that enables us to rigorously investigate and predict the magnetoresistive properties by simulations. Using hybrid spin dynamics and molecular dynamics simulations we first study the structuring process of interacting magnetic nanoparticles in the gel matrix. Based on the resulting nanoparticle arrangements we calculate the corresponding characteristic magnetoresistive curves by means of spin dynamics simulations. With this procedure, detailed information on the magnetoresistive properties of arbitrary nanoparticle arrangements can be predicted. In addition to that, an intensive interaction between numerical and experimental investigation was established. 3D reconstructions of images taken with a dual system composed of a focused ion beam (FIB) and a scanning electron microscope (SEM) give detailed information about particle sizes and positions in real systems. This information can directly be used for spin dynamics simulations which allow to calculate the microscopic magnetic and magnetoresistive properties. Besides the sensitivity of a sensor device which is dominated by the magnetoresistive behavior, the performance of the sensor surface plays an important role. Here, we present a method to determine the association and dissociation constants in molecular recognition based on the sensor signal taken for the PhoB binding processes to DNA as a biological example.

## 2. Interplay of Microstructure and Magnetoresistive Characteristics of Nanoparticular Systems

In order to design magnetoresistive sensor devices, one needs detailed insight into microstructure and mechanisms that are involved. For this purpose, a strong entanglement of experimental investigation and simulation is required. Here, we present numerical achievements alongside experimental methods with the objective of improving the comprehension of fundamental mechanisms leading to beneficial magnetoresistive properties.

### 2.1. Towards a Rational Design Tool for Magnetoresistive Sensor Devices by Means of Hybrid Molecular Dynamics and Spin Dynamics Simulations

The spatial arrangement of the magnetic nanoparticles in the gel matrix is mainly responsible for the magnetoresistive properties of the sensor device. Hence, detailed information about the structuring process driven by external magnetic fields is necessary. Classical spin dynamics (SD) is a numerical method which allows to compute the static and dynamic magnetic properties of a spin system as a function of temperature. It can be applied to microscopic and mesoscopic ensembles of classical magnetic moments. In the following we assume single domain nanoparticles, *i.e.*, an effective magnetic moment is assigned to each particle, related to its diameter and saturation magnetization [6]. In addition to that, we assume that exchange interactions between particles do not play a role and therefore only magnetic dipole dipole interaction is taken into account. We used a SD algorithm where the heat bath contact is modeled using a Langevin approach. Details concerning the algorithm can be found in [7]. Such SD simulations assume fixed positions of the magnetic moments and determine the orientations of the magnetic moments by solving the spin equations of motion. However, in the liquid gel, the magnetic forces due to inter-particle interactions and external fields lead to particle movement that has to be addressed by a second simulation method, namely molecular dynamics (MD). MD is a numerical method that computes trajectories of classical point particles, *i.e.*, dimensionless particles which are set in motion by forces that are derived from classical potentials. The simulation of nanoparticles that interact by magnetic dipole dipole interaction and which are free to move due to magnetic forces is only possible by following a hybrid molecular and spin dynamics approach. For the MD part of our simulation, the highly-specialized software package HOOMD-blue [8] has been chosen because of its remarkable range of available functions. In order to include the magnetic dipole dipole interaction in the MD code, a potential energy contribution according to Equation (1) is evaluated for every particle pair in the simulated system for every step in time:

(1) $${ℋ}_{DD}=-\frac{{µ}_{0}}{4\pi {\left|r_{12}\right|}^{3}}\left[3\left(m_{1}\cdot \frac{r_{12}}{\left|r_{12}\right|}\right)\left(m_{2}\cdot \frac{r_{12}}{\left|r_{12}\right|}\right)-m_{1}\cdot m_{2}\right]$$

Within this equation, $\left|r_{12}\right|$ denotes the distance between the magnetic moments $m_{1}$ and $m_{2}$ of the particles 1 and 2, while $r_{12}$ represents the vector that joins the centers of particles 1 and 2. The usage of point particles in HOOMD-blue in combination with magnetic dipole dipole interaction poses a general problem for our hybrid approach that has to be addressed first. The magnetic dipolar forces exhibit a singularity that occurs when the particle distance approaches zero. In order to avoid such singularities we have implemented the so-called Weeks-Chandler-Anderson potential [9] that provides a short-range repulsion so that the simulated particles behave like hard spheres that interact by their magnetic dipolar fields:

(2) $${ℋ}_{WCA}=\{\begin{array}{l} 4\varepsilon \left[{\left(\frac{\sigma }{\left|r_{12}\right|}\right)}^{12}-{\left(\frac{\sigma }{\left|r_{12}\right|}\right)}^{6}\right]+\varepsilon ,\;if\;\left|r_{12}\right|<\;2^{\frac{1}{6}}\sigma \\ 0,\;if\;\left|\;\right|\;\geq \;2^{\frac{1}{6}}\sigma \end{array}$$

Therein, ε represents the depth of the attractive potential well, given in energy units and σ, given in units of distance, denotes the distance at which the potential becomes zero. The potential is cut off at a distance of $2^{\frac{1}{6}}\sigma$. By choosing ε=σ=1, the simulated particles behave like hard spheres with given diameters. Similarly to the magnetic dipole dipole interaction, the WCA potential is evaluated for every particle pair in every step in time.

**Figure 1:**
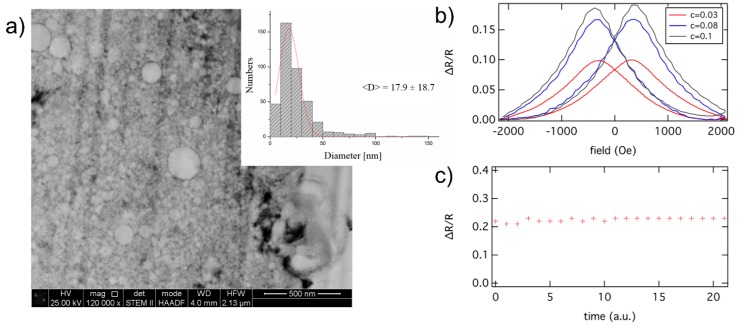
GMR effect of cobalt particles embedded in agarose. (**a**) Particle size distribution of Co core/conductive C-shell nanoparticles used for magnetoresistive measurements in agarose matrix utilizing an AC mode; (**b**) Experimentally achieved GMR effect as a function of particle concentration c which is defined by the ratio of particle mass over gel mass. The GMR effect amplitude is given in the ratio of ΔR to R with R, the electrical resistance, which relates to an effect amplitude of about 10% to 20%. The reliability of an effect amplitude of above 20% is given in (**c**) where the GMR effect of a sample has been repeated for 20 times.

In general, a hybrid MD and SD approach requires to solve the equations of motion simultaneously. For our hybrid approach we assumed that the translational degrees of freedom for each nanoparticle are much slower than its magnetic degrees of freedom, *i.e.*, the change of the orientation of the magnetic moment. This allows us to decouple the calculation of the degrees of freedom reminiscent of the Born-Oppenheimer approximation [10]. Starting with an initial configuration consisting of the positions and magnetic moment orientations for each nanoparticle, we first use SD in order to compute the magnetic ground state for that configuration. After that, the new magnetic moment orientations are used in the MD part to compute the corresponding forces and integrate the equations of motion for a predefined number of time steps, resulting in new positions which are again passed over to the SD calculation. These steps are repeated alternately until the maximum number of time steps is reached. We applied our method to a sample of 192 cobalt nanoparticles with diameters of 20 nm which is chosen to closely mimic the experimental situation which is depictured in Figure 1. In our model, the particles are placed in an area of 350 nm × 350 nm. Therefore, 2 × 10^7^ hybrid MD/SD time steps have been performed with one full SD run every 1 × 10^5^ time steps. In zero external magnetic fields, the formation of chain fragments and small islands containing magnetic vortices can be observed. During the structuring process, the magnetic dipolar energy and the total magnetization are gradually reduced until a minimum has been reached.

**Figure 2:**
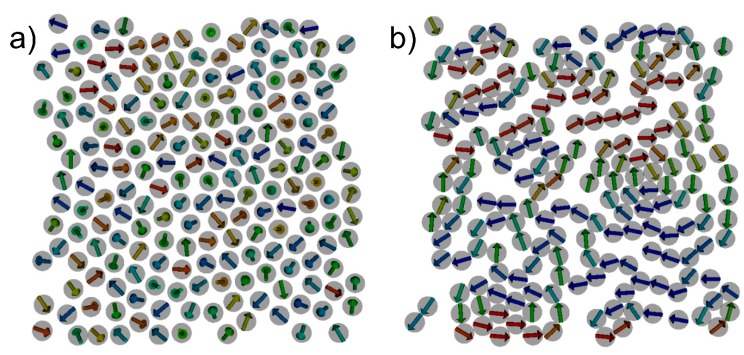
Result of hybrid SD-MD simulation in zero external fields for a test structure containing 192 Co particles. The formation of chains and islands containing magnetic vortices can be found which is in agreement with the experimental observations. The total magnetic energy decreases from E_mag_ = −1.6512E−13 J for the initial state (**a**) to E_mag_ = −3.34852E−15 J for the final state (**b**).

**Figure 3:**
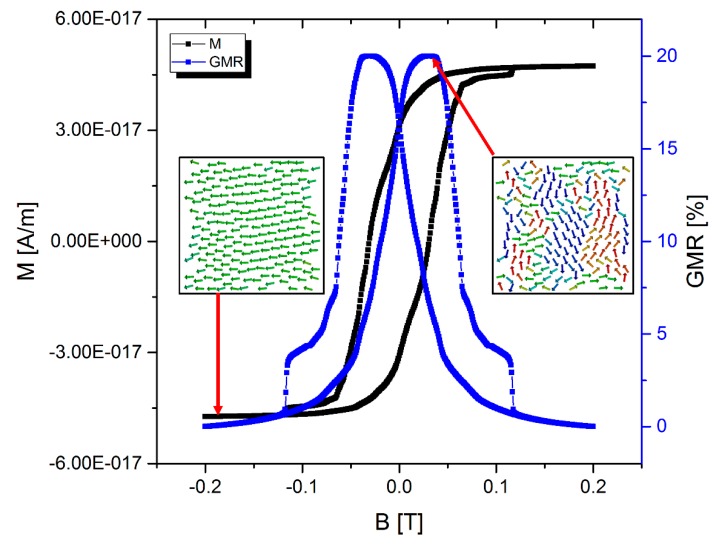
Calculated GMR curve for our SD/MD-optimized sample of 192 Co particles. The magnetization curve (black dots) has been obtained by means of spin dynamics simulations after performing a complete hybrid SD-MD simulation. From the magnetization data, the qualitative GMR curve (blue squares) can be extracted according to Equation (3). For the sake of simplicity, the GMR effect amplitude is assumed to be 20%, according to the experimental results shown in Figure 1.

This situation corresponds to a “freezing” with respect to the positions and the orientations of the magnetic moments as well. The initial configuration and the final configuration of the particles are shown in Figure 2. In analogy to experiments, the next step is to dry out the gel matrix in order to fix the positions of the nanoparticles. In the simulation, this can be done by simply switching off the MD part and just perform SD simulations. In order to compare to experimental results [5] we have to determine the magnetoresistive properties. Experimentally, the electrical resistance of such structures can be measured with a four-point-probe setup [5] and the GMR is determined by measuring the resistance for different external magnetic fields. In the framework of SD simulations the GMR characteristics can be derived from the total magnetization according to [11,12]:

(3) $$GMR=A_{GMR}\left[1-{\left(\frac{M}{M_{S}}\right)}^{2}\right]$$

with *M*, the magnetization and *M_S_*, the saturation magnetization. The GMR effect amplitude $A_{GMR}\;$ can be calculated by means of quantum mechanical techniques [13] or it can be determined experimentally. We matched $A_{GMR}=20\%$ to our experimental data. Figure 3 shows a calculated magnetization curve for the structure under consideration obtained by means of classical spin dynamics simulations and the resulting GMR curve according to Equation (3).

### 2.2. Efficient Calculation of Low Energy Configurations of Frustrated Dipolar Systems

**Figure 4:**
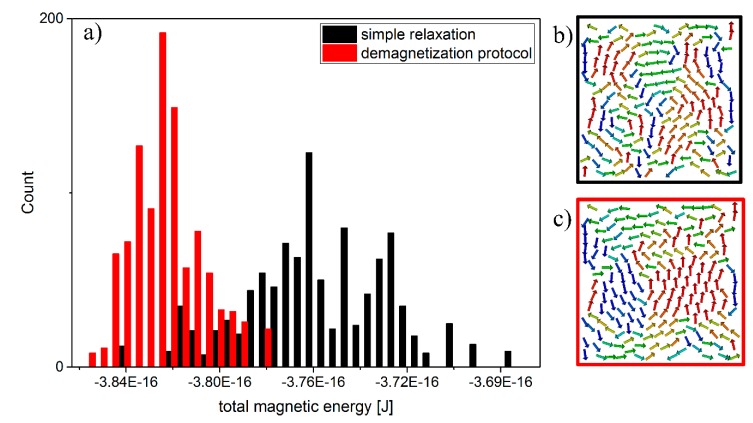
Evaluation of a total of 1000 simulations following a simple relaxation procedure and 1000 simulations using our demagnetization protocol resulting in lower total magnetic energies and a narrower distribution (**a**); Application of a demagnetization protocol leads to energetically favorable configurations with larger vortices (**c**) compared to the simple relaxation procedure (**b**); The colors of the arrows in (b,c) indicate the x-component of the magnetic moment of each particle.

After gelatinization of the gel matrix, the particle positions are fixed in space. The magnetic state of a rigid configuration can be investigated by means of SD. Simulations at T = 0 K reveal a sophisticated behavior due to the dipolar coupling between the particles and their spatial disorder. Instead of one unique ground state, a multitude of low energy configurations has been found. Here, the inherent magnetic frustration of the particle arrangements due to the dipole dipole interaction poses computational challenges reminiscent to those of calculating the properties of large-scale spin glass systems [14]. A standard relaxation algorithm fails to predict a global energy minimum because the system gets trapped in local energy minima that are separated by energy barriers. In order to solve that problem we have implemented a demagnetization routine [6] that has been successfully applied to the numerical investigation of artificial spin ice [15] which belongs to a class of spin systems that is very similar to our system. In our demagnetization routine the sample is exposed to a sinusoidally varying, rotating, and damped magnetic field. Using this technique one can overcome the energy barriers which occur due to strong inter-particle interactions with the result that lowest energy configurations can be found much more efficiently as compared to the standard relaxation algorithm. Results of simulations obtained by a standard relaxation and simulations using our demagnetization routine are shown in Figure 4. For the demagnetization routine, 30 sinusoidal oscillations, 7 azimuthal, and 3 polar turns have been applied during a simulation run of 1 × 10^−7^ s while damping the magnetic field from 0.05 T to 0 T.

### 2.3. Combining Experiment and Simulation: Spin Dynamics Simulations of 3D Reconstructed Sensor Systems

**Figure 5:**
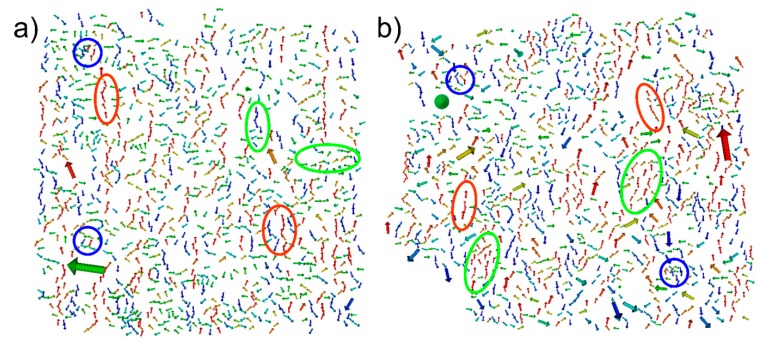
Results of SD simulations of two experimentally investigated structures, one prepared in a homogeneous external magnetic field (**a**) and one prepared in a rotational field (**b**). The particle positions and sizes were obtained by 3D reconstruction of images taken by a dual FIB-SEM system. The length and thickness of the arrows indicates the size of the magnetic moment, whereas the color indicates the x-component of the magnetic moment. The simulations reveal vortex-like structures (blue), ferromagnetically ordered (green), and chain-like (red) areas.

The GMR effect amplitude depends on several parameters including the volume fraction of particles along the current path and the overall particle concentration. The microstructural arrangement of magnetic nanoparticles can be investigated using a dual system composed of a focused ion beam (FIB) and a scanning electron microscope (SEM). For a detailed study, the dual method was applied along with SD simulations. With the dual system, SEM images are taken while cutting away thin slices of the sample successively. Afterwards a three-dimensional reconstruction of the SEM images provides detailed topological information of the magnetic nanoparticle distribution, whereas the magnetoresistive properties can be determined by a four-point probe method with the contacting needles configured in-line as proposed in [5]. To gain insight into the mechanisms that control the magnetoresistive properties, detailed information about the microscopic magnetic structure is necessary, which can be obtained by performing SD simulations. This method has been applied to two samples consisting of cobalt nanoparticles in an agarose matrix. In order to perform the dual FIB-SEM experiments, the sample was freeze dried whereby the agarose matrix is losing its conductivity. Thus, the sample was made conductive by placing a thin gold layer on top. By extracting the particles’ positions and sizes from 3D reconstructions and using the information as input for SD simulations, it can be shown that potential sensor configuration are composed of particle chains, vortices and ferromagnetically ordered areas, as shown in Figure 5. Because the structure exhibits strong geometric frustration, the numerical technique described in Section 2.2 has been applied. By comparing the magnetoresistive and magneto-structural information, it can be shown that the strong ferromagnetic order of closely packed particles decreases the GMR effect amplitude, whereas antiferromagnetically ordered chains increase the GMR effect (details on magnetoresistive characteristics will be published elsewhere). Moreover, information about the GMR characteristics can be extracted from simulation data, as described in Section 2.1. As a result, the combination of the experimental dual FIB-SEM technique and numerical spin dynamics simulations provides a powerful tool for the investigation of magnetoresistive effects in detail and it is crucial for the rational development of future sensor devices. By combining SD and MD simulations to one hybrid method and making use of improved SD for frustrated systems, together with experiments done by the dual FIB-SEM method, a very elaborate tool chain has been created. With this, one is able to gain detailed insight in the formation of the sensor’s microstructure and can investigate the role of different parameters on its magnetoresistive properties.

## 3. Modeling the Binding Process between Two Biological Molecules Based on the Sensor Signal

The generic application of a magnetoresistive sensor is to quantify the rate of coverage for biomolecules (*A*) on the sensor surface which is determined by a change in magnetoresistivity. Experimentally, this change can be detected when these *A* biomolecules are labeled with superparamagnetic beads. The *A* biomolecules bind to partner biomolecules (*B*) within a biocoating of the sensor surface. While bound to the surface, the beads that are attached to the *A* biomolecules are in close proximity to the sensor and their magnetic stray field gives rise to a change in magnetoresistance, as soon as an external magnetic field is applied [11].

That way, the magnetoresistive sensor is capable of measuring the concentration of *A* biomolecules which are bound to the sensor surface. Hence, depending on the strength of the binding between *A* and *B* biomolecules a surface concentration profile as a function of time is expected which is defined by the association rate *k_a_* and dissociation rate *k_d_* of the two molecule partners. Consequently, the question arises whether it is possible to determine both rate constants in order to gain insights into the binding process. This will enable one to quantify the binding efficiency which provides additional important biological information. We have developed a transient model which allows to model the capture process of any bioactivated sensor surface, as long as it is a simple 1:1 binding between the corresponding molecules *A* and *B*. The equations describing these processes are solved using finite element simulations with COMSOL Multiphysics^®^ [16].

### 3.1. Development of the Transient Model

**Figure 6:**
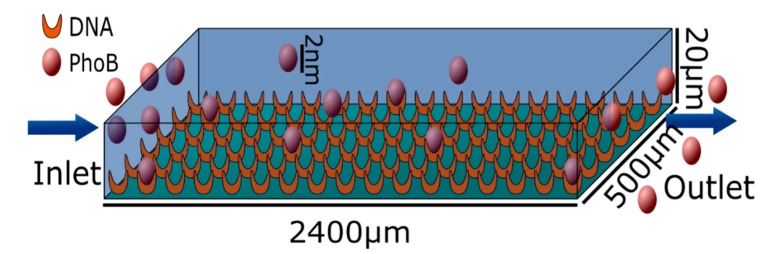
Geometry of the flow cell where the PhoB is detected. The bottom of the cell is covered with DNA which binds to the PhoB protein. Water is flowing from the left side to the right.

**Figure 7:**
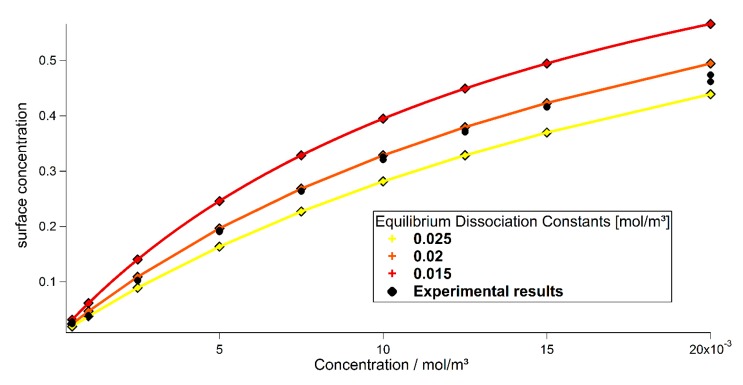
Concentration dependency of the surface concentration of bound proteins with respect to the equilibrium dissociation constant $K_{D}$. Experimental results (black, taken from [18]) motivated the theoretical values (yellow, orange, red).

The model geometry used for our tests is shown in Figure 6. A cuboid that measures 2400 × 500 × 20 μm resembles the sensor surface used in [17]. All other parameters were chosen to reproduce the behavior of the wildtype PhoB binding to the DNA coated sensor surface. PhoB is a phosphate regulon transcriptional regulatory protein [17,18]. The hydrodynamic radius of PhoB is estimated to be $r_{\text{PhoB}}\approx 2\text{nm}$ and the diffusion coefficient is, assuming the fluid to be water at 20 °C, D ≈ 10^−10^ m^2^∙s^−1^. The mean velocity of the fluid flow is $u=3.3\;\text{cm}\cdot {\text{s}}^{-1}$, the density of the DNA acceptors on the surface is estimated to be $n_{A}=2.56\cdot 10^{-8}\;\text{mol}\cdot {\text{m}}^{-2}$, the inlet concentrations are $c_{in}\in \left\{0.5,\;1,\;2.5,\;5,\;7.5,\;10,\;12.5,\;15,\;20\right\}\cdot 10^{-3}\text{ mol}\cdot {\text{m}}^{-3}$. The equilibrium dissociation constant is according to [17] $K_{D}\approx 21\cdot 10^{-3}\text{ mol}\cdot {\text{m}}^{-3}$ (see Figure 7). The dissociation constant $k_{d}$ is estimated to be about $\;k_{d}\approx 1.5\cdot 10^{-8}\text{ mol}\cdot {\text{m}}^{-2}\cdot {\text{s}}^{-1}$. This value has been taken from the experimental investigations published [17] which are used here to validate our model. Both dissociation constants were used to calculate the association constant $k_{a}$:

(4) $$k_{a}=\frac{k_{d}}{K_{D}}\approx 7.14\cdot 10^{-7}\text{m}\cdot {\text{s}}^{-1}$$

The main transport mechanism is the convection through the fluid’s movement. Therefore, the first simulation step is to solve the Stokes equation and the equation of continuity:

(5) ∇p=ηΔu  ,  ∇⋅u=0

with the pressure difference ∇p, the fluids viscosity η and the velocity vector u. All boundaries except the inlet and outlet are no-slip boundaries, which are described by  u=0. Inlet and outlet have a fixed pressure. As PhoB is a small protein, the diffusion constant is rather large and the gravitational force may be neglected. Thus, the transport equation for the proteins is:

(6) $$\frac{\partial c}{\partial t}+D\text{Δ}c-\nabla \text{c}\cdot u=0$$

with c being the protein concentration. The outlet is described as –N⋅D∇c=0, the walls and the top of the channel are of the no flux type condition N⋅( uc−D∇c)=0, with N being the inward normal vector. The sensor is set to a Robin type boundary condition, as it is described in [19]:

(7) $$N\cdot D\nabla c\;=\;-k_{a}c(1-\frac{n}{n_{A}})+k_{d}\frac{n}{n_{A}}$$

where n is the surface concentration of proteins bound to the sensor’s surface. As the surface concentration changes over time, an ordinary differential equation is defined for the surface, to count the proteins bound to the surface. This process is described by:

(8) $$\frac{\partial n\;}{\partial t}\;=\;k_{a}c(1-\frac{n}{n_{A}})-k_{d}\frac{n}{n_{A}}$$

with an initial value of n(0)=0. The initial value for the concentration is c(x,0)=0. The concentration at the inlet is smoothly raised from 0 to $c_{in}$ in 0.5 s. The system is subjected to this concentration for 20 s, thereafter the concentration of PhoB proteins in the water is reduced to 0 in another 0.5 s.

### 3.2. Results and Calculation of Rate Constants

**Figure 8:**
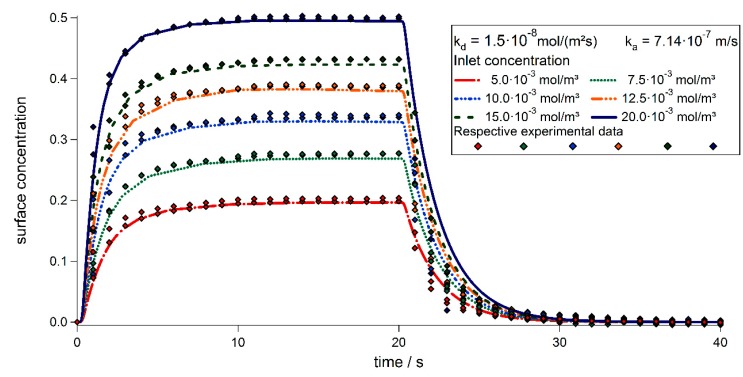
Comparison between simulation results of PhoB binding to the sensor surface (similar to [17] Figure 3f) with respect on different inlet concentrations obtained from our model to experimental data from [19]. The time scale of the experimental data was adjusted in order to match the experimental and theoretical time scales.

**Figure 9:**
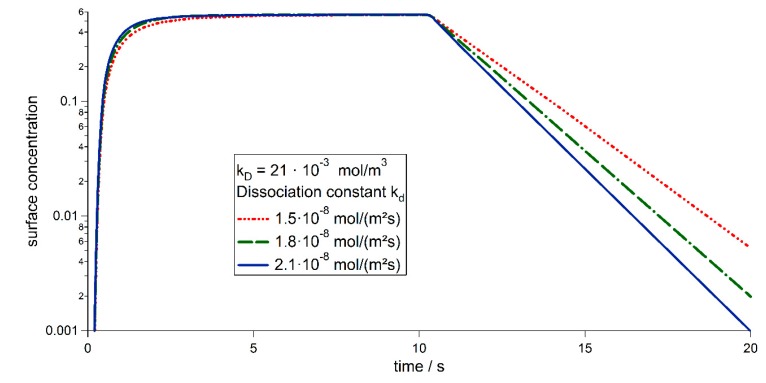
Results for the simulation of PhoB binding to the sensor surface (similar to [17] Figure 3f). Shown is the dependence on the dissociation constant *k_d_* for a fixed equilibrium dissociation constant *K_D_* and a concentration of $c_{in}=20\cdot 10^{-3}\text{mol}\cdot {\text{m}}^{-3}$. The surface concentration is plotted in a logarithmic scale, to point out the exponential decay of the surface concentration.

Our simulations shown in Figure 8 are in good agreement with experimental results [17], since both, association and dissociation are close to the ones in the experiment. The peak concentration of bound particles shows the same behavior as the experimental data and consequently the same equilibrium dissociation constant could be derived as shown in Figure 9. For the equilibrium state, where both, on and off rates are the same, Equation (8) may be rewritten to the equation for the one-site binding model used in [17,18]. Therefore, the concentration of the proteins may be derived from the surface concentration utilizing the equilibrium dissociation constant *K_D_*. Figure 9 shows the solution for a concentration of $c_{in}=20\times 10^{-3}\text{mol}\cdot {\text{m}}^{-3}$ and three different dissociation constants *k_d_* on a logarithmic scale. After the concentration is switched off at 20 s (red line) the bound particle concentration decreases exponentially with three well distinguishable slopes. An exponential fit can be utilized to give an estimate for the dissociation constant for the experiments conducted in [17]. The association constant cannot be derived directly from the experimental data. However, knowing *k_d_* and *K_D_*, it may be calculated using Equation (4). This evaluation may be used, as long as the dissociation is fast enough to be measured during the measuring process. When working with very slow dissociation rates, the association process and its corresponding constant may be estimated using the model presented. The model may also be extended to treat samples which are subjected to buoyancy, the transport equation then has to be extended as described in [20].

## 4. Conclusions/Outlook

Magnetoresistive sensor devices that are composed of magnetic particles in conductive matrices instead of conventional multilayer systems show promising features concerning potential sensor sensitivities. We have presented a tool chain that allows to investigate these systems numerically in combination with an experimental technique. Experimentally obtained configurations of magnetic nanoparticles are used as input for simulations that reveal the magnetic microstructure which is not directly accessible by experiments. In doing so, detailed information about the interrelation of the structuring process and resulting magnetoresistive properties can be achieved. We presented a novel hybrid simulation technique that simultaneously treats magnetic and kinetic degrees of freedom of systems of interacting magnetic particles. Based on the resulting structures qualitative GMR curves can be calculated and hence, a first evaluation of the magnetoresistive properties of a system can be performed from scratch. Moreover, the role of magnetoresistive sensors in finding answers to molecular recognition has been investigated so as to determine the association and dissociations rates of biomolecules covering the magnetoresistive sensors surface. We have presented finite element simulations that reveal information about the transient association rate and dissociation rate in a system assuming a 1:1 binding which is compared to the experimentally determined binding of PhoB proteins to a DNA functionalized surface. With this example, we built a bridge between the fundamentals of magnetoresistive sensor devices and a possible application in the field of molecular recognition.
